# 

**Published:** 1907-08-24

**Authors:** 


### BOOKS RECEIVED.

E. Marlborough and Co.

"Travellers' Practical Manual of Conversation."

T. Nelson and Sons.

"The History of David Grieve." By Mrs. Humphry Ward.
"The King's Mirror." By Anthony Hope. Nelson Library.
"John Charity." (Nelson Library.)
Great Eastern Railway Co.'s Tourist Guide to the Continent.

H. Frowde and Hodder and Stoughton.

"Auscultation and Percussion." By S. Gee, M.D.
"Heart Disease and Thoracic Aneurysm." By F. J. Poynton, M.D.
"Clinical Lectures and Addresses on Surgery." By C. B. Lockwood.
"Operations of General Practice." By E. M. Corner, M.A., and H. Irving Pinches, M.A.
"Diseases of the Male Generative Organs." By E. M. Corner, M.A., M.B.
"Prostatic Enlargement." By Cuthbert S. Wallace, M.B.
"A System of Medicine." Edited by W. Osler, M.D., and T. McCrae, M.D. Vol. II.

J. Clarke and Co.

"Health in the Home Life." By Honnor Morten.

W. B. Clive.

"Matriculation Directory," June 1907.

J. B. Lippincott Co.

"International Clinics," 17th series, Vol. II.

J. Wright and Co.

"A Skin Pharmacopœia." By J. Startin.

"Through Jamaica with a Kodak." By Alfred Leader.

### General Practitioners' Contributions.

**Important.**

We propose to devote a special page to General Practitioners' Contributions. We therefore invite from practitioners contributions based upon their experience in the management of cases, and in the treatment and diagnosis of disease; especially shall we be prepared to welcome articles dealing, practically, with treatment, and with the use and value of new remedies and methods.

No article should exceed 1,100 words in length, and, if accepted, one guinea will be paid to the writer after publication. Each communication should be accompanied by a stamped directed envelope for the return of the MS. if found unsuitable.

**The Relaxations of Medical Men.**

We shall also be glad to pay for accepted contributions, from any member of the profession, on the subject of the relaxations of practitioners. This opens up a wide field, as it includes natural history, photography, sport, indoor recreations, and motoring. Whenever possible, original illustrations and photographs should be sent with the MS.

**Suggestions Invited.**

The Editor will welcome suggestions for the establishment of any new section in The Hospital, and will be glad to supply information on any subject of interest or importance to members of the profession in any part of the world.

### Notices and Answers to Correspondence.

All MSS., letters, books for review, and other matters intended for the Editor, should be addressed to The Editor, The Hospital Building, 28 and 29 Southampton Street, Strand, London, W.C.

THE HOSPITAL *August* 24, 1907.

*Name* ..........................................................................

*Address* ..........................................................................

..........................................................................

This Coupon must accompany manuscript or contributions intended for The Hospital.

